# Bioelectric networks: the cognitive glue enabling evolutionary scaling from physiology to mind

**DOI:** 10.1007/s10071-023-01780-3

**Published:** 2023-05-19

**Authors:** Michael Levin

**Affiliations:** 1 Allen Discovery Center at Tufts University, 200 Boston Ave., Suite 4600, Medford, MA 02155 USA; 2 Wyss Institute for Biologically Inspired Engineering at Harvard University, Boston, MA USA

**Keywords:** Morphogenesis, Voltage, Development, Regeneration, Basal cognition, Evolution, Behavior, Cells

## Abstract

Each of us made the remarkable journey from mere matter to mind: starting life as a quiescent oocyte (“just chemistry and physics”), and slowly, gradually, becoming an adult human with complex metacognitive processes, hopes, and dreams. In addition, even though we feel ourselves to be a unified, single Self, distinct from the emergent dynamics of termite mounds and other swarms, the reality is that all intelligence is collective intelligence: each of us consists of a huge number of cells working together to generate a coherent cognitive being with goals, preferences, and memories that belong to the whole and not to its parts. Basal cognition is the quest to understand how Mind scales—how large numbers of competent subunits can work together to become intelligences that expand the scale of their possible goals. Crucially, the remarkable trick of turning homeostatic, cell-level physiological competencies into large-scale behavioral intelligences is not limited to the electrical dynamics of the brain. Evolution was using bioelectric signaling long before neurons and muscles appeared, to solve the problem of creating and repairing complex bodies. In this Perspective, I review the deep symmetry between the intelligence of developmental morphogenesis and that of classical behavior. I describe the highly conserved mechanisms that enable the collective intelligence of cells to implement regulative embryogenesis, regeneration, and cancer suppression. I sketch the story of an evolutionary pivot that repurposed the algorithms and cellular machinery that enable navigation of morphospace into the behavioral navigation of the 3D world which we so readily recognize as intelligence. Understanding the bioelectric dynamics that underlie construction of complex bodies and brains provides an essential path to understanding the natural evolution, and bioengineered design, of diverse intelligences within and beyond the phylogenetic history of Earth.

## Introduction: from cells to cognition in the collective intelligence of organisms

**Fig. 1:**
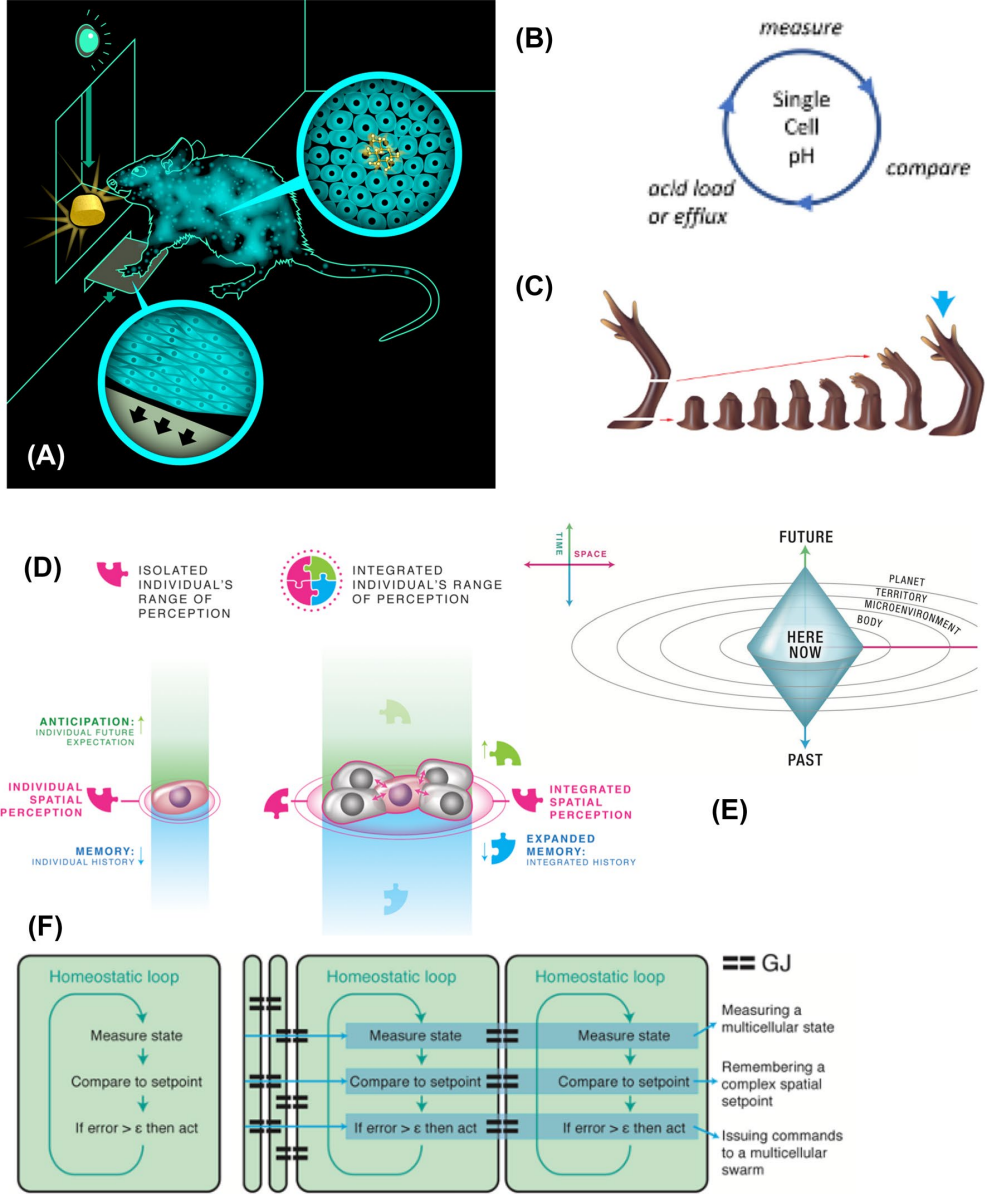
Cognitive scaling. **A** Rat learning to press a lever to get a reward illustrates the principle of cognitive scaling: the rat is a collective of cells, some of which interact with the lever (skin of the paws) and some of which get the nutrient reward (intestinal cells). However, no single cell has both experiences, and it’s the causal structure of the tissue network that enables credit assignment for behaviors, so that an associative memory between an action and a reward can form that belongs to the whole animal and none of its parts alone. **B** Simple homeostatic cycle (in this case, for pH control) indicative of the kinds of low-level goals that single cells can pursue. **C** Anatomical homeostasis, such as reliably regenerating toward a complete, correct salamander limb regardless of where it is cut, and stopping when that goal is achieved, is an example of large-scale (organ-level) collective behavior that emerges from the activity of a network of cells. **D** By merging together into computational networks, cells (neural or other) can increase the spatial scale of sensing, temporal memory and predictive power (sensing backward and forward in time, respectively). **E** Every agent has a “cognitive light cone” which demarcates the spatio-temporal scale of the goals toward which it can expend energy. This cone changes during evolution and during the lifetime of an animal from that of a single cell, with single-cell scale goals, to that of a morphogenetic system with organ-level goals and eventually a behavioral system with large-scale goals in 3D space. **F** This scaling of behavioral competencies and goal states in a given problem space can be implemented by gap junction (GJ) connections, which enable a connection of homeostats into a network that scales up the setpoint, measurement, and action steps of evolutionarily ancient homeostatic loops. All images created by Jeremy Guay of Peregrine Creative, used with permission. **B**-**F** taken with permission from (Levin 2022)

All animals with advanced conventional cognitive capacities are the products of a very lengthy evolutionary process of accumulating gradual modifications of early microbial life. Even more remarkably, each of us personally took a journey in which matter acquired mind: we start life as a quiescent oocyte, and slowly remodel into an adult modern human with metacognitive, self-aware capacities and the ability to reason about entire counterfactual universes. How did the information processing capabilities of a single cell (Lyon 2006, 2015), with metabolic and physiological competencies, inflate to that of a human, who can pursue goals of planetary scale whose endpoint may be far after their expected lifespan (Fig. 1)? Taking evolution, and especially developmental biology, seriously means coming to grips with the continuous process of transformation that enables configurations of matter to express increasingly greater degrees and kinds of cognitive capabilities: decision-making, generalization, perception, causality detection, innate responses, learning, and advanced problem-solving. The emerging field of *basal cognition* (Baluška and Levin 2016; Levin et al. 2021; Lyon et al. 2021) strives to understand the evolutionary and embryological origins of our current behavioral capacities, and uncover the physical and computational dynamics by which simple minds emerge from chemistry and scale over time.

**Fig. 2:**
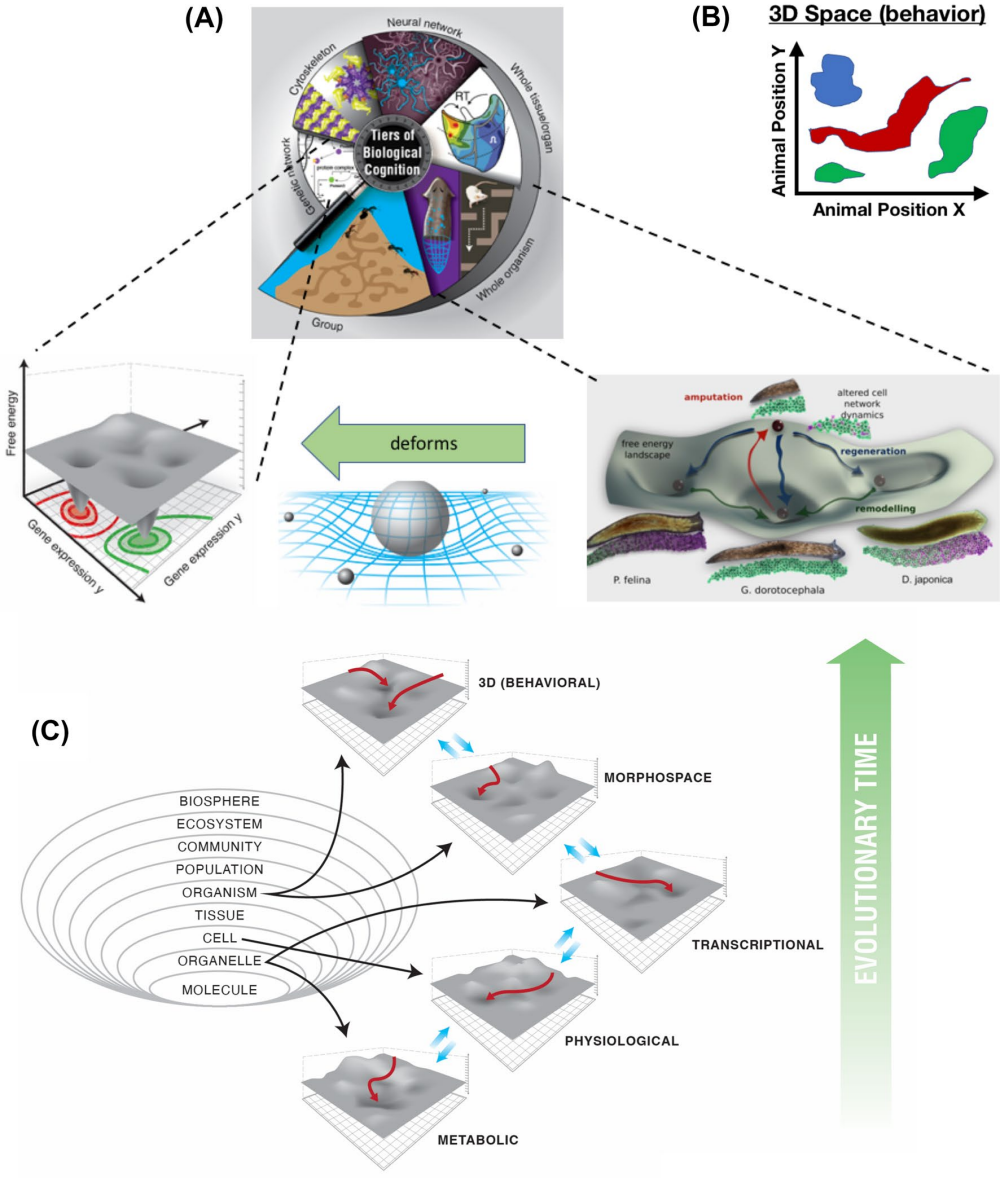
Multiscale competency architecture operates across problem spaces. **A** Biological systems are nested dolls in which molecular networks give rise to subcellular components, such as cytoskeletal networks, which give rise to cells, tissues, organs, organisms, and swarms. This multiscale architecture is not only structural, but rather functional: each level solves problems (with some degree of competency) in its own action space. Each level deforms the energy landscape for the levels below and above, influencing those components to do things they would otherwise not do. For example, when traversing the morphospace of different planarian head shapes, the bioelectric circuit controlling head morphogenesis alters the space of gene expression that is necessary to implement the specific anatomical outcomes. **B** Familiar (conventional) behavior is navigation of a three-dimensional space, in which animal positions move to optimize certain reward functions. Although the human visual and cognitive repertoire is most accustomed to recognizing competency of typical (mid-sized, animal) agents navigating three-dimensional spaces, this deep concept integrates ideas across such wide-ranging fields as autonomous robotics (AI), evolutionary fitness landscapes, morphospaces, and cognitive behavioral studies. **C** There are in fact many problem spaces in which biological systems operate. Evolution progressively pivoted existing and novel mechanisms across problem spaces, to enable adaptive navigation toward specific goal states in metabolic, physiological, transcriptional, morphological, and ultimately behavioral space. All images created by Jeremy Guay of Peregrine Creative, except for the planaria image of panel **A**, which was created by Alexis Pietak. Used with permission; **A**,**B** taken with permission from (Levin 2022)

It is essential to expand the scope of this inquiry beyond traditional metrics of intelligence and behavior (Fig. 2). As humans, we are very good at recognizing intelligence of medium-sized objects moving at medium speeds in three-dimensional space: our sense organs face outward, and our capacities for detecting agency in this action space are strong (Mar et al. 2007; Repp and Knoblich 2007). However, assigning an IQ estimate to any being or object is, in effect, taking an IQ test ourselves: it is easy to miss intelligent behavior if one cannot recognize it. Imagine if we had a well-developed internal sense of our own body chemistry states—if we had the equivalent of a tongue that faced inwards into the bloodstream, it would then be natural for us to recognize all the remarkably clever things our liver and kidneys were doing with respect to managing us in the high-dimensional space of body physiology. Could recognizing *diverse intelligence* (in unfamiliar substrates) be essential to understanding the mechanisms and origins of conventional cognition and behavior?

William James anticipated a cybernetic approach to this problem by defining intelligence as a degree of “the ability to reach the same goal by different means” (James 1890). This definition is not limited to brains or specific kinds of behaviors—it is intentionally agnostic about the composition of the agent. It challenges us to define a problem space, for any arbitrary system (including unconventional embodiments), and then formulate and test specific hypotheses about the competencies that the system can deploy in navigating that space. Traditional concepts of reflex responses, drives, memory, different kinds of learning, goal-directed activity, and even higher level capacities such as planning and creativity can all be defined in other problem spaces beyond the familiar 3D space, including metabolic, physiological, transcriptional, and anatomical spaces.

It has previously been proposed that the evolutionary path to conventional cognition involves evolutionary pivots across these problem spaces (Fields and Levin 2022). This suggests a broadening of the traditional systems to which these cognitive terms apply, and asking what is *essential* about them that is deeper than the specific frozen accidents that the course of terrestrial evolution has provided in its *N* = 1 history of life on Earth (Clawson and Levin 2022). Here, through the lens of a gradualist, evolutionary perspective I explore the possible origins of cognitive capacities and the extension of these concepts to diverse embodiments.

The goal is not to review the steps of evolution of electrically excitable networks (Arendt et al. 2016; Brunet and Arendt 2016; Jekely 2019; Jekely et al. 2015; Keijzer and Arnellos 2017; Keijzer 2017; Levin et al. 2021; Lyon et al. 2021). Rather, I aim to provide a novel perspective on how to understand animal behavior in the broader context of “control of agential activity” in all its diverse guises. A deep unification is within reach, if we can find and exploit invariants—symmetries between a variety of processes and biological systems that shed light on generic principles for scaling of cognition and collective intelligence. In fact, all intelligence is collective intelligence—not just termite mounds and beehives. We too are emergent beings supervening on a collection of cells which all were once independent unicellular organisms. How do they work together to enable the creation of a novel being with goals and memories that belong to it and not to any of its parts? The key question is not only the scale-up of a unitary cognitive capacity, but the many-into-one transition: the emergence of minds from collectives (Levin 2019, 2021b), working in spaces beyond those of their component cells.

At stake is not only a better understanding of our evolutionary history, but also insight into the relationship between genome and functional forms, and strategies for modifying, improving, and building novel synthetic cognitive agents (with implications ranging from exobiology to regenerative medicine). The over-arching framework (Levin 2022) is fundamentally grounded in the continuity hypothesis, using biophysical and evolutionary approaches to identify invariants and symmetries across natural evolved forms, as well as hybrid and fully synthetic life forms (and perhaps, some day, truly alien beings) (Fields and Levin 2022; Levin 2022). Here, I illustrate one segment of this framework’s roadmap by focusing on one highly instructive example: developmental bioelectricity as a precursor of brain-like processes, which reveals not only evolutionary pivots between two different problem spaces, but also shows a path to solving the problem of collective intelligence across scales of organization. In this discussion, I intentionally avoid issues of first-person experiential consciousness, focusing instead on third-person observable capacities for behavior broadly. I describe how computation via voltage states enabled coordinate navigation of anatomical morphospace before brains and muscles enabled us to navigate 3D space, as an example of how familiar behavioral science concepts can be generalized to gain insight into the origin and mechanisms of cognition. Here, cognition is not restricted to advanced capabilities such as planning, self-aware metacognition, language, etc., but is considered broadly in accordance with its continuous developmental and evolutionary origins. It serves as an umbrella term for *all* degrees of adaptive information processing and problem-solving (Levin 2022), no matter how advanced or primitive. The focus on bioelectricity below, as a kind of tractable cognitive glue, is one facet of a broader emerging research program designed to help detect, understand, and relate to a wide range of natural, artificial, and hybrid intelligences regardless of size scale or material implementation (Abramson and Levin 2021; Calvo et al. 2020; Fields et al. 2021; Fields and Levin 2022; Ginsburg and Jablonka 2021; Gokhale et al. 2021; Kuchling et al. 2020; Levin 2021b, 2022; Lyon 2019; Ramstead et al. 2022; Smith-Ferguson and Beekman 2020; Timsit and Grégoire 2021; Watson et al. 2022).

## Beyond standard model organisms: stories of plasticity and change

**Fig. 3:**
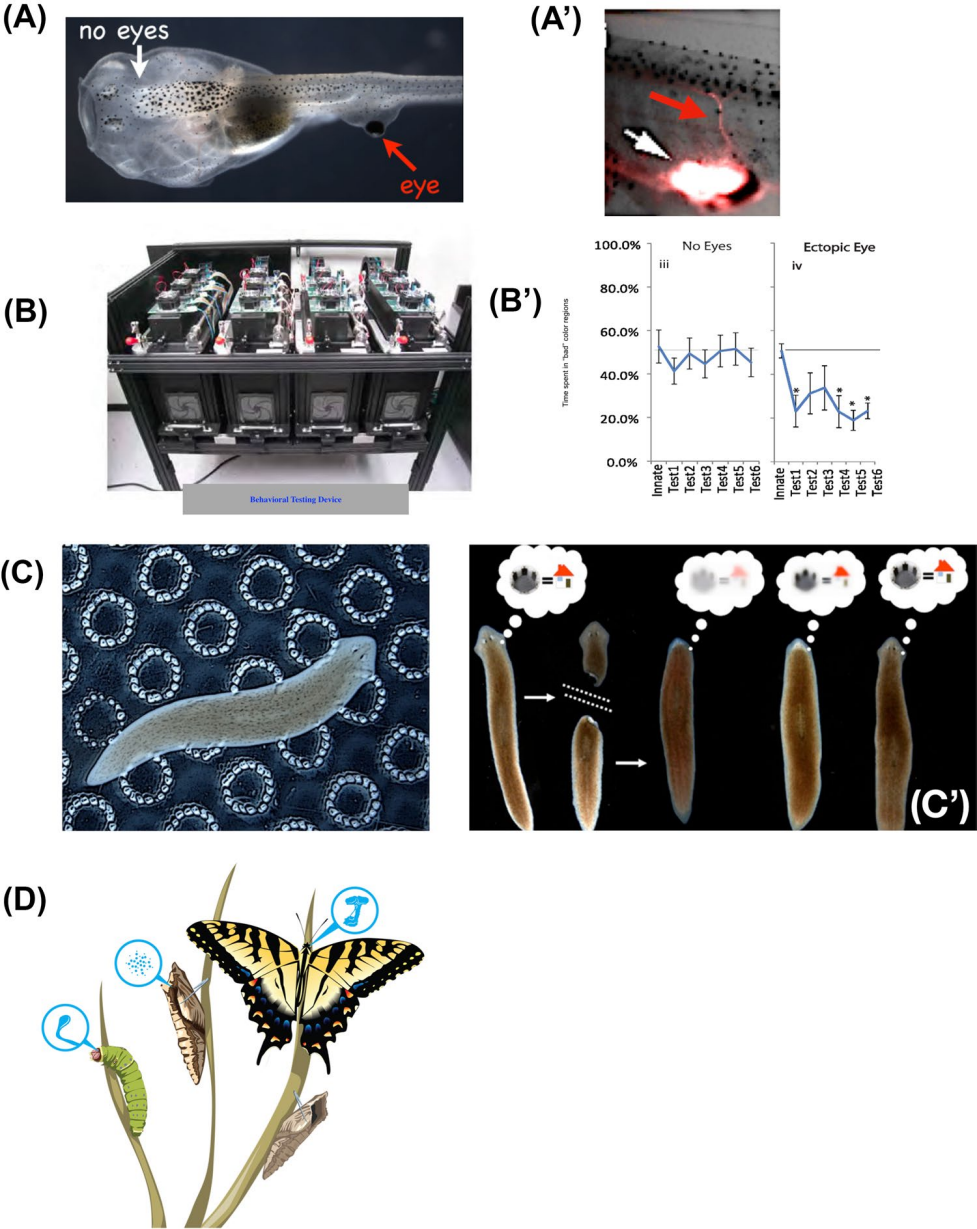
Unconventional agents: plasticity and robustness to change. **A** Tadpole of the frog *Xenopus laevis* can be made to have no primary eyes (white arrow), but instead have an ectopic eye on its tail (red arrow). **A’** These ectopic eyes (white arrow) can connect to the spinal cord (red arrow). **B** Using an automated behavioral training and testing apparatus, these animals can be shown to be able to see out of those eyes in a color vision training assay (**B’**) despite a novel visual system architecture that had no evolutionary adaptation—a remarkable example of functional plasticity despite wild-type genetics. **C** Planarian flatworms can be trained to associate laser-etched circular regions (bumps) in a petri dish surface with food. When their heads are amputated (**C’**), their behavior shows recall of the original information (place conditioning), showing the ability of memory to be stored outside the head and imprinted on newly produced brain tissue (showing how functional, behavioral memories are dynamically integrated with the tissue-level patterning processes that create specific shapes in anatomical morphospace). **D** Caterpillars (and other insect larvae) metamorphose into very different forms, which requires extensive disassembly and rebuilding of the brain. Despite this, their memories persist, showing that individual agents change during their lifetime not only due to experiences and learning, but also can radically change with respect to anatomical structure. Panel **D** created by Jeremy Guay of Peregrine Creative. Panels **A**–**C** used by permission from (Blackiston et al. 2010; Blackiston and Levin 2013; Levin 2022; Shomrat and Levin 2013); **C’**,**D** used by permission from (Levin 2022). (color figure online)

To begin to broaden the inquiry into the evolutionary origin and mechanisms of conventional cognition, it is important to consider several biological examples that stretch standard assumptions (Fig. 3). First, consider *Physarum*: a unicellular slime mold, which has been used as a popular model for basal cognition (Beekman and Latty 2015; Nakagaki and Guy 2007; Reid et al. 2016; Saigusa et al. 2008; Vallverdú et al. 2018). Recent work has shown that this organism can exhibit learning following repeated experience, eventually becoming willing to cross areas of noxious chemicals to receive a reward (Boisseau et al. 2016; Boussard et al. 2019; Vogel and Dussutour 2016). Moreover, when placed in an arena containing distant inert glass objects of different mass distributions, it uses a biomechanical mechanism to process information about its environment and then reliably grow towards the heavier object (Murugan et al. 2021). These examples of learning and decision-making occur in the absence of a brain, neurons, or cellularization. From the perspective of evolutionary change across behavioral domains, one interesting thing is that for *Physarum*, its behavior *is* its morphological change*.* In this system, changing body shape to exploit the environment is how it implements behavior. Distinctions between morphological problems and behavioral problems are blurred by the biology, and the classical (Cartesian) conceptual distinctions between mind and body, which lead to separate communities for developmental biology and behavioral science, are now increasingly seen as artificial, for example, in the field of morphological computation in robotics (Bongard and Levin 2021).

It has been proposed that the mechanisms of memory establishment are the same as those which sculpt brain tissue developmentally (Galván 2010; Kandel and O'Dell 1992). Planaria offer another example, where anatomical and behavioral information are tightly linked (Saló et al. 2009). These free-living flatworms, with a true centralized, bilaterian brain (Pagán 2014; Sarnat and Netsky 2002), have the ability to regenerate their bodies—every piece of a planarian gives rise to a properly patterned new worm (Sheiman and Kreshchenko 2015). However, this process not only implements an anatomical memory of body structure. Tails of worms trained on specific tasks regenerate into animals that show recall of the original information: the behavioral memories are apparently also imprinted onto the new brain as it forms (McConnell et al. 1959; Shomrat and Levin 2013). Thus, the information processing required to restore a specific body shape and that required to propagate the results of past experience despite cellular turnover and maintenance are tightly linked.

Metamorphosis tells a similar story. Caterpillars must become moths or butterflies, requiring turning a controller that operates a soft body in a two-dimensional lifestyle into one that operates a hard body in a three-dimensional world. The brain is largely dismantled and rebuilt in a new configuration, but learned information persists (Alloway 1972; Blackiston et al. 2008; Sheiman and Tiras 1996). Such dynamic plasticity is not just for invertebrates. Tadpoles of the frog *Xenopus laevis* can be produced with no primary eyes, but one eye on their tails; these animals can see out of an ectopic eye, which can connect to the spinal cord (Blackiston and Levin 2013). In one generation, requiring no evolutionary adaptation, the cellular hardware of a frog embryo can adapt to this radical reconfiguration of its visual sensory system.

Thus, radical changes of behavioral repertoires occur not only on evolutionary time-scales, but also at the level of an individual being—paralleling embryogenesis in raising profound questions about the *transformation of agents by rearrangements of their parts*—a key aspect of recognizing even traditional animals as fundamentally collective intelligences. A key aspect of understanding both basal and traditional cognition is formulating paradigms for predicting the properties and capabilities of emergent Selves from those of the components that comprise them.

This is fundamentally a story of the multi-scale competency architecture that biology employs. Animals are nested dolls made up of cells and tissues but this arrangement is not merely structural; cells were once unicellular organisms and have many competencies in their own problem spaces [an agential material which evolution molds by behavior-shaping as much as changes in their hardware (Davies and Levin 2023)]. When one “trains a rat” to obtain a treat by pressing a lever, the cells that interact with the lever (skin) are not the same cells that obtain the metabolic reward (intestine). No single cell had both experiences, and the owner of the associative memory linking those two events is an emergent collective intelligence (Fig. 1A). Likewise, even gene regulatory networks can learn relationships between their experiences (such as Pavlovian conditioning) as a collective, by virtue of the activity of very simple components (transcriptional elements) (Biswas et al. 2021; Watson et al. 2010). Conversely, the voluntary act of raising one’s arm reveals the functional connection between the highest levels of executive function to the depolarization of muscle cells—information crossing levels from that of human thoughts to the physiological status of individual cells. What is the coordination mechanism that enables this cross-level integrated information processing? It is developmental bioelectricity.

## Bioelectricity: the ancient cognitive glue

**Fig. 4:**
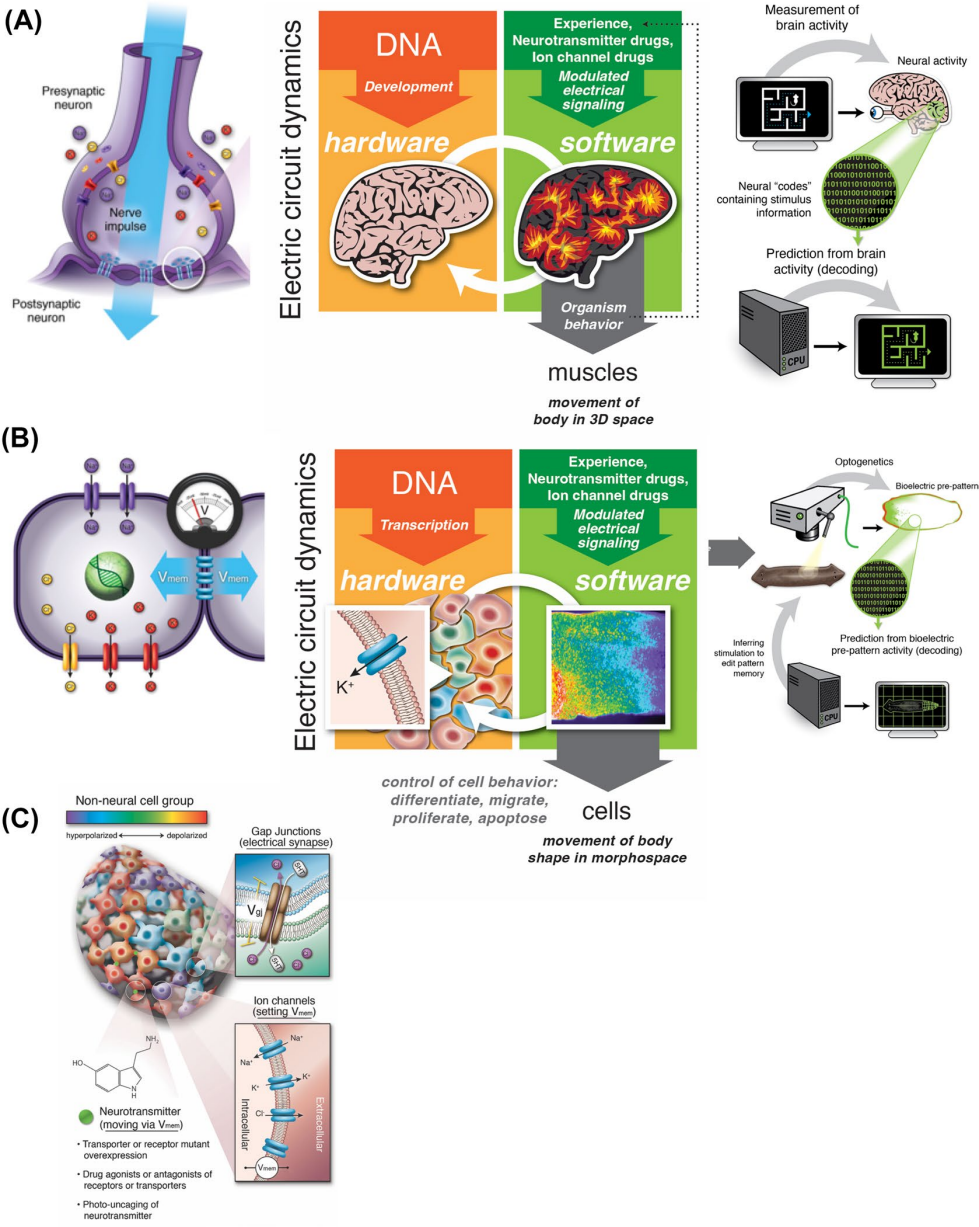
Developmental and neural bioelectricity: a deep symmetry. **A** Familiar hardware of neurons consists of ion channels in the membrane which set voltage state, and electrical synapses (gap junctions) which enable those states to selectively propagate through the network. This enables a kind of software phenomenon—physiological events that process information and guide behavior. The effort of neural decoding is the practical implication of the idea that all of the agent’s memories, plans, preferences, behavioral repertoires, etc. are instantiated in that bioelectrical layer of control and will be able to be read out (interpreted) once we understand the encoding. **B** Exact same architecture is used throughout the body, and forms the evolutionary precursor of the behavioral control system. All cells have ion channels, and most cells couple via regulated gap junctions to their neighbors, enabling the bioelectric physiology that guides growth and form during morphogenesis. Consistent with the evolutionary pivot model, these electrical networks also process information to enable navigation: prior to navigating 3D space by controlling muscle action (when brains appeared), this system was used to process information and make decisions, while bodies navigated anatomical morphospace during embryogenesis, regeneration, and cancer suppression. **C** This isomorphism between somatic and neural bioelectricity is what enables all of the tools of neuroscience to be used outside of the brain. Developmental bioelectricity is studied by voltage imaging dyes, and functional techniques such as genetic, chemical, and optical ways of regulating ion channels and gap junctions in vivo. The tools (and many concepts) are broadly compatible across tissue types, enabling the insights of behavioral neuroscience to be portable toward understanding outcomes in other problem spaces. All images created by Jeremy Guay of Peregrine Creative and used with permission; **A**,**B** taken with permission from (Levin 2022)

The functional properties of brains and nervous systems emerge from a network architecture in which neurons (and other cells like glia) communicate via changes in cellular resting potential (*V*_mem_) and the resulting movement of neurotransmitter signals. *V*_mem_, the bioelectric state of each nervous system component, is determined by an integrated balance of charges via the action of ion channels and pumps that enable the segregation of potassium, sodium, chloride, and protons (Fig. 4). Bioelectric states can propagate between cells via electrochemical synapses known as gap junctions (GJs) (Mathews and Levin 2017; Palacios-Prado and Bukauskas 2009). GJs form direct connections between the cellular internal milieus that enable the transfer of current and small chemical messengers. In addition, bioelectric states can also induce the movement of neurotransmitter molecules, such as serotonin, both through the extracellular space (via transporters and vesicles) and directly through gap junctions (Levin et al. 2006; Romero-Reyes et al. 2021). Crucially, every component in this network is functionally regulated by every other, and by its own action: ion channels and gap junctions are themselves often voltage-sensitive (Brink 2000; Palacios-Prado and Bukauskas 2009), while neurotransmitters move under electromotive force and regulation, and in turn regulate ion channel properties.

This extremely rich set of feedback loops establishes computational capacity; for example, ion channels and GJs, as voltage-gated current conductances, are in effect transistors and possess a fundamental property of historicity (memory in which past events impact current signaling state). These events do eventually impact other kinds of pathways (such as gene expression), but it is critical that the information processing in such networks is essentially physiological—the rapid propagation of signals via action potentials and slow waves across the network does not itself require transcriptional change. As a corollary, the information content of this network cannot be read out at the transcriptional or even proteomic level: channels open and close post-translationally, and the same channels can give rise to different voltage states depending on cells’ history, while diverse channels can give rise to the exact same voltage map.

There is no one-to-one mapping between the molecular state and the bioelectrical state, making it essential to study such systems in the living condition (unlike genetic and protein-level information, which can be studied in fractionated or fixed material, bioelectrical information disappears at cellular death). One implication of this feature is a critical separation of hardware and software (Boone and Piccinini 2016). Of course, the ability to form stable behavioral repertoires based on specific kinds of past experience, and the way in which it coarse-grains and generalizes from a sensory stream, are shaped by the network’s structure and physical properties. However, structurally identical networks can have learned different things after physiological experiences—bioelectric networks’ historicity means that their information content is not hardwired by their genetic specification but is dependent on past experience. One cannot know the informational content of a brain merely from knowing its neural layout and genome: the exact same brain can contain numerous different memories, goals, etc. This decoupling of the material state (protein content) from the information content is the first step to the most amazing aspect of neural networks: they enable mind to arise from matter. Specifically, neural networks are the functional layer in which physiology transitions to meaning: electrophysiological events encode memories, plans, preferences, behavioral capacities, and a first-person perspective—the content and shape of a cognitive Self, at whatever level of sophistication.

Neuroscience [via the research program of neural decoding (Huth et al. 2016; Naselaris et al. 2009; Nishimoto et al. 2011)] is committed to the idea that all of the content of minds, from the most primitive to the most complex, can be read out from (and thus resides in) the electrophysiological state of the network. Thus, just as inorganic electric circuits and transistors enable the jump from the physics of Ohm’s law to the truth tables of logic gates (basic elements of formal thought), biological electric circuits and ion channels/GJs enable the jump from chemistry to embodied meaning. A crucial aspect of this is that it provides a scaling up of agency: the homeostatic competencies of single neurons are integrated into a network that supports an emergent, higher order Self with memories, preferences, and other features that belong to itself but not to any of its components individually. Bioelectric networks, with their inherent plasticity, multiscale historicity, and learning capacity, are an ideal kind of “cognitive glue” that binds the primitive goal-directedness (in the cybernetic sense) of single cells into a higher order system with a larger cognitive light cone (Fig. 1E). However, perhaps, the most fascinating and far-reaching aspect is that the key features that enable the supervenience of active information on a material substrate are not unique to brains and nervous systems at all (Fig. 4).

The use of bioelectric networks to enable coherent computation to occur via a spatially and temporally distributed living medium is ancient, both in its molecular components (which date back to our unicellular ancestors) and in the algorithms by which it provides adaptive function. Even bacterial biofilms use electrical networks to synchronize activity within the proto-body of the colony (Martinez-Corral et al. 2019; Prindle et al. 2015; Yang et al. 2020). Evolution discovered long ago that networks made up of ion channels, gap junctions, and neurotransmitters as transduction machinery for electric circuit function provide a remarkably powerful and flexible way to process information. The current use of these components to implement behaviors in 3D space by controlling muscle activity represents an evolutionary pivot: their original usage was to navigate anatomical morphospace (Fields et al. 2020) (that is, to control all cell behaviors toward specific morphogenetic outcomes). This conservation of both molecular mechanisms and algorithms is the reason that the workhorse tools of neuroscience work everywhere in the body—they do not distinguish between neuronal and non-neuronal uses. Clear morphogenetic analogs (Pezzulo and Levin 2015) exist for optogenetics, ion channel mutants, neurotransmitter drugs—the tools of neuroscience—as well as for neuroscience concepts, such as memory, representation, navigation, perceptual bistability, and many others (Table 1).

**Table 1:** Conceptual mapping between behavioral cognition and anatomical regulation

Cognition	Morphogenesis
Action potential movement within an axon	Differential patterns of *V*_mem_ across single cells’ surface
Local field potential (EEG)	*V*_mem_ distribution of cell group
Intrinsic plasticity	Change of ion channel expression based on *V*_mem_ levels
Synaptic plasticity	Change of cell:cell connectivity via *V*_mem_’s regulation of gap junctional connectivity
Activity-dependent transcriptional changes	Bioelectric signals’ regulating gene expression during patterning
Neuromodulation	Developmental (pre-nervous) signaling via neurotransmitters such as serotonin moving under control of bioelectrical gradients
Direct transmission	Cell:cell sharing of voltage via nanotubes or gap junctions
Volume transmission	Cell:cell communication via ion levels outside the membrane or voltage-dependent neurotransmitter release
Synaptic vesicles	Exosomes
Sensitization	Cells become sensitized to BMP antagonists to stabilize neurogenesis
Functional lateralization	Left–right asymmetry of body organs
Taste and olfactory perception	Morphogenetic signaling by diffusible biochemical ligands
Activity-dependent modification of CNS	Control of anatomy by bioelectric signaling within those same cells
Critical plasticity periods	Competency windows for developmental induction events
Autonomic reflexes	Wound healing
Voluntary movement	Remodeling, regeneration, metamorphosis
Memory	Shorter term: regeneration of specific body organs. Longer term: morphological homeostasis over decades as individual cells senesce; altering basic body anatomy in planaria by direct manipulation of bioelectric circuit
Generalization via multi-layer neural networks	Generalization by bow-tie architectures of signaling pathways
Pattern completion ability of neural networks (e.g., attractor nets)	Regeneration of missing parts in partial fragments (e.g., planaria)
Forgetting	Cancer, loss of regenerative ability
Addiction	Limb becomes unable to regenerate without nerve once exposed to nerve
Encoding	Representation of patterning goal states by bioelectric properties of tissue
Perceptual bi-stability	Stochastic flipping between two target morphologies by planarian fragments in the cryptic state
McGurk effect	Modification of interpretation of biochemical signals by bioelectric state of cells
Visual system feature detection	Organ-level decision-making during morphogenesis
Mirror neurons and mirror focus in epilepsy	Contralateral bioelectric signals mirroring sites of amputation damage in frog legs
Holographic (distributed) storage	Any small piece of a planarian remembers the correct pattern (even if it has been re-written)
Instinct	Hardwired patterning programs (mosaic development)
Behavioral plasticity	Regulative developmental programs and regenerative capacity
Self-modeling	Surveillance of anatomical state by brain
Goal-seeking	Embryogenesis and regeneration work towards a specific target configuration despite perturbations
Sub-goaling in problem solving tasks	Developmental modularity
Adaptivity and intelligence	Morphological rearrangements carrying out novel, not hardwired, movements to reach the same anatomical configuration despite unpredictable initial starting state
*Tabula rasa*	Cells could be a (semi) universal constructor, able to build any shape that can be specified via the pattern memory code
Attention and context salience	Tissues responding to specific bioelectric states only when relevant (e.g., when in damage state)
Age-dependent cognitive decline	Age-dependent loss of regenerative ability
Optogenetic insertion of false memories	Optogenetic induction of regeneration or ectopic organs
Reading of semantic content from brain scans	Detecting differences in target morphology from fluorescent voltage dye data
Executive control (free action) filtering down to regulate muscle motion	Large-scale circuit decisions that dictate whole bodyplan axial patterning transduced to individual cell gene expression changes as needed to implement large-scale phenotype

Conceptual mapping of ideas and phenomena between neuro-behavioral sciences to morphogenesis (listed in rough order of ascending levels of organization). These are discussed in more detail elsewhere (Pezzulo and Levin 2015, 2016).

The bioelectric system is so versatile that it was readily exapted for behavior when nerve and muscle evolved (Keijzer et al. 2013), with two major changes: a significant speed-up (milliseconds, instead of hours, as the primary time scale) and a focus on *temporal* signaling (spiking patterns) for behavior instead of development’s reliance on *spatial* bioelectric patterns across tissues. Despite these differences, profound symmetries between the problems of morphogenesis and the problems of cognition exist; remarkably, while only recently explored in detail, this idea was already obvious to Alan Turing as early as 1952 (Turing 1952) and to others in the following decades (Grossberg 1978). Much as electrophysiological neural events are the fundamental currency that underlies the emergence of a coherent behavioral Self with some degree of cognitive activity, their slower, ancient ancestor mechanisms enable individual cells to cooperate toward coherent anatomical goals and deploy problem-solving capabilities in morphospace that belong to the “embryo”—an emergent collective.

## Basal cognition without neurons: what dynamic bodies think about

**Fig. 5:**
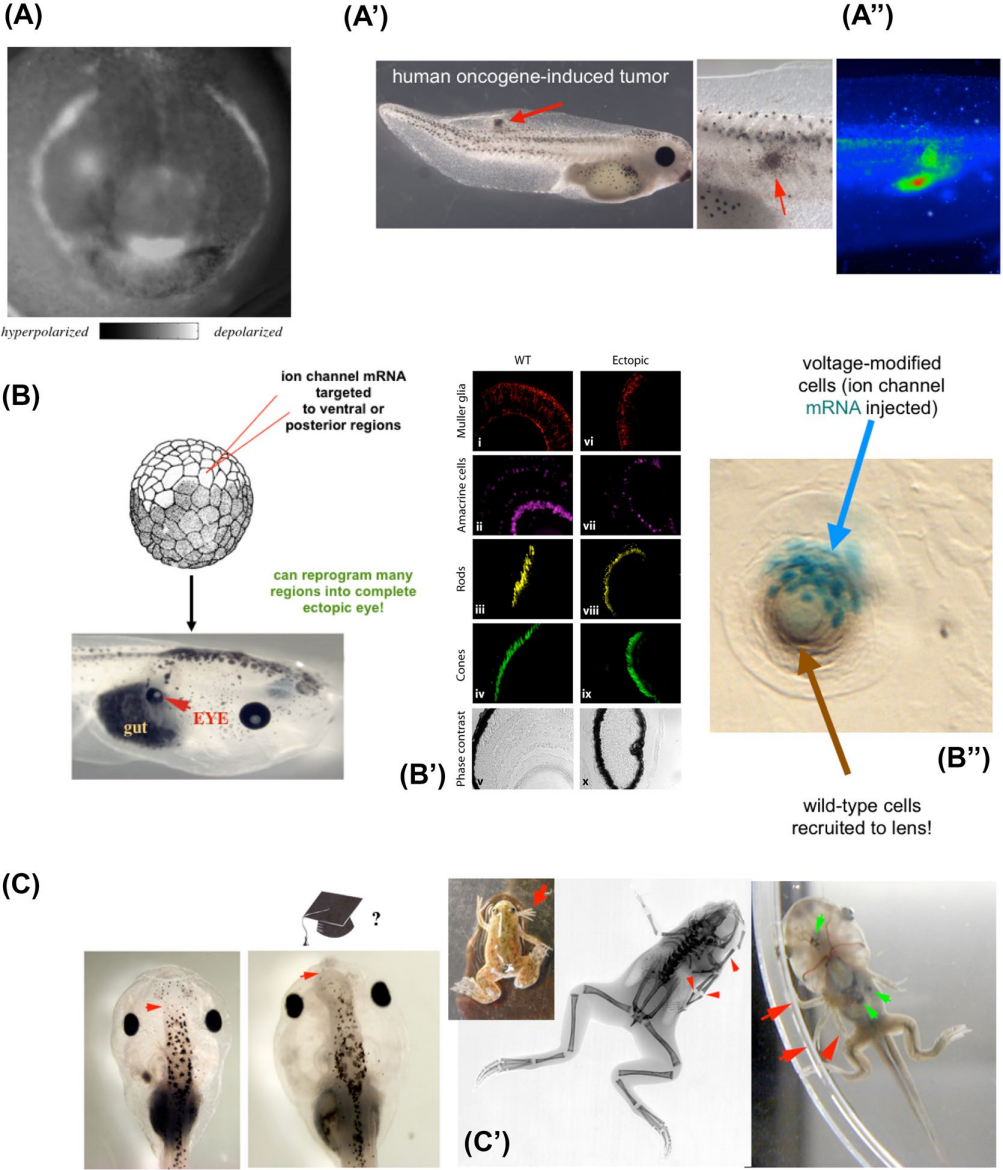
Examples of non-neural bioelectricity guiding behavior in morphospace. **A** Much as brain imaging allows the reading of bioelectrical states in tissues to decode the properties of control circuits and behavioral competencies, voltage reporter dyes enable in vivo tracking of the information processing in morphogenetic decision-making. Here is shown one frame from a timelapse video of a frog embryo prior to formation of the face, showing a prepattern of resting potential states that demarcates the position of the future gene expression domains and craniofacial organs, such as the eyes, mouth, and lateral structures. In contrast to this endogenous pattern, pathological patterns (such as those leading to tumors in A’) can be induced via, for example, oncogene injection. The location of tumors (A”, red arrowhead) can be predicted by the bioelectric dye signal which shows an aberrant electrical signature of cells that are disconnecting from the tissue-level network and reverting back to unicellular-scale behavior (i.e., metastasis and over-proliferation). **B** These bioelectric prepatterns are known to be instructive, because reproducing them elsewhere by misexpression of specific ion-channel mRNA, such as in this frog embryo, results in induction of whole organs, such as eyes (red arrow), which have the necessary internal tissue structure (immunohistochemistry in **B’**). This demonstrates a key aspect of behavior—binding complex downstream actions to a simple (low information-content) trigger. Moreover, this phenomenon exhibits the competency of recruitment (**B”**): when only a few cells are injected with the channel (cyan b-galactosidase marker label), they autonomously recruit normal neighbors to complete their morphological goal: building a normal-sized ectopic lens (brown tissue). **C** Method of inducing whole organs (control of large-scale movements in anatomical morphospace) by modulating bioelectric states of the tissue circuit (i.e., incepting false memories into the network) can also, for example, produce ectopic forebrain (red arrow), or ectopic limbs (**C’**, **C”**, red arrows). Panels reused with permission from (Chernet and Levin 2013a; Levin 2009; Levin et al. 2017; Pai et al. 2012; Vandenberg et al. 2011). (color figure online)

**Fig. 6:**
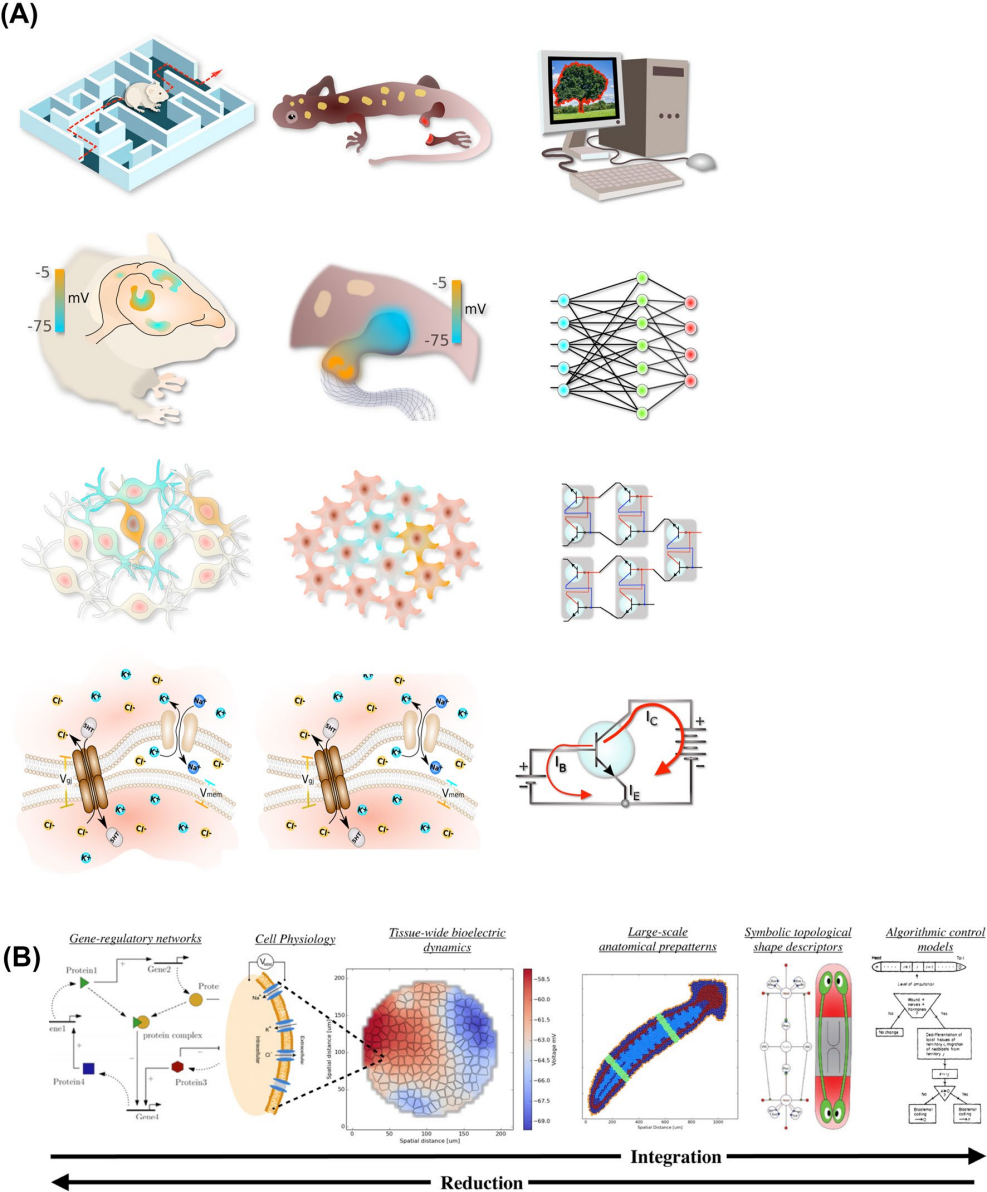
Isomorphism between neuroscience, morphogenesis, and artificial intelligence. **A** Multiscale nature of information-processing architectures are shown, with the left column (neuroscience), middle column (morphogenesis), and right column (computer engineering). In each case (going down from top row), high-level phenomena such as behavior and morphological competencies are mediated by real-time physiological dynamics that enable decision-making and learning, which operate on electrical networks that perform computations, which in turn are enabled by molecular machinery that implements voltage-gated current conductors (ion channels and gap junctions in the case of cells, and transistors in the case of digital computers). **B** In behavioral science, the full panoply of phenomena requires understanding of molecular synaptic and ion channel machinery, circuit functions, network capabilities, behavioral repertoires, and eventually executive-level goal-directed activity. Similarly, in developmental bioelectricity, the field aims for a “full stack” integration of transcriptional networks that drive ion channel expression, to tissue-level voltage dynamics, to organ-level decisions about size and shape, ultimately to high-level algorithms that control the axial bodyplan and organ layout. Images by Alexis Pietak, used by permission

**Fig. 7:**
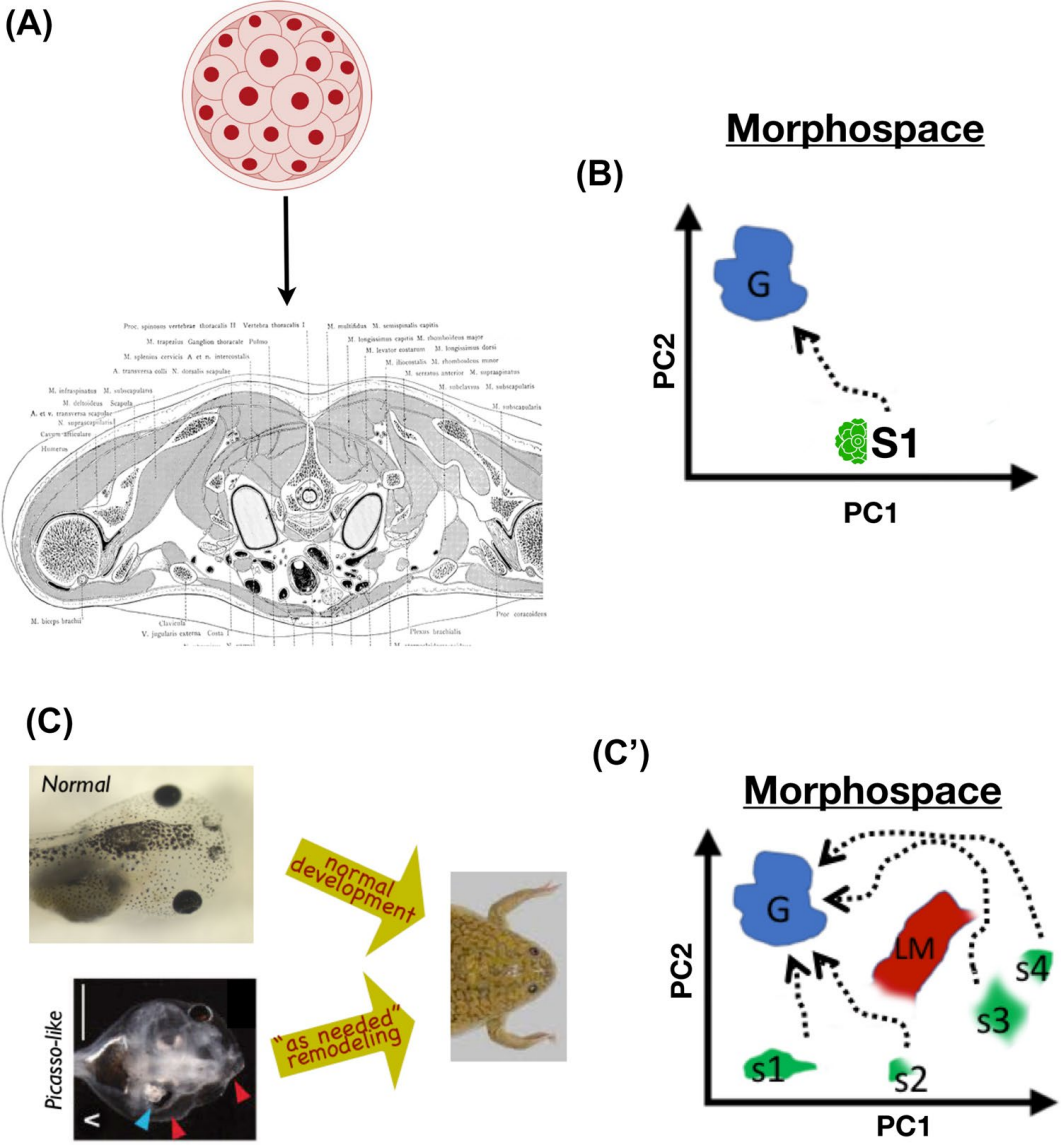
Morphogenesis as competent behavioral navigation. **A** Animals begin life as a single cell and then a ball of embryonic blastomeres, which eventually gives rise to the incredible complexity of the body (cross section of the human torso is shown here). **B** Viewing anatomical structure as a morphospace of parameters describing possible configurations (here simplified to 2 dimensions, principal components (PC) 1 and 2), one can imagine this process as a hardwired transition from a starting state (S1) to an ensemble of states corresponding to viable adult organisms (goal states G). **C** However, when probed by perturbation (as any novel animal’s behavior is studied), traversals of morphospace are revealed to exhibit considerable flexibility. Here is shown an example of metamorphosis in the frog *Xenopus laevis*. Not only do normal tadpoles (starting from a standard, correct state) become frogs by rearranging the components of their face, but so do “Picasso tadpoles” in which all the organ positions have been scrambled. This is because the organs then move in novel paths and different distances, *as needed*, to achieve a normal frog craniofacial morphology, thus showing the capacity (**C’**) to move to the right region of morphospace from diverse starting positions (S1–S4) and despite various obstacles (local minima LM, in which less competent navigational agents would get stuck). Panels **A**,**B**,**C’** by Jeremy Guay of Peregrine Creative; **C** courtesy of Douglas Blackiston and Erin Switzer, and taken with permission from (Vandenberg et al. 2011)

**Fig. 8:**
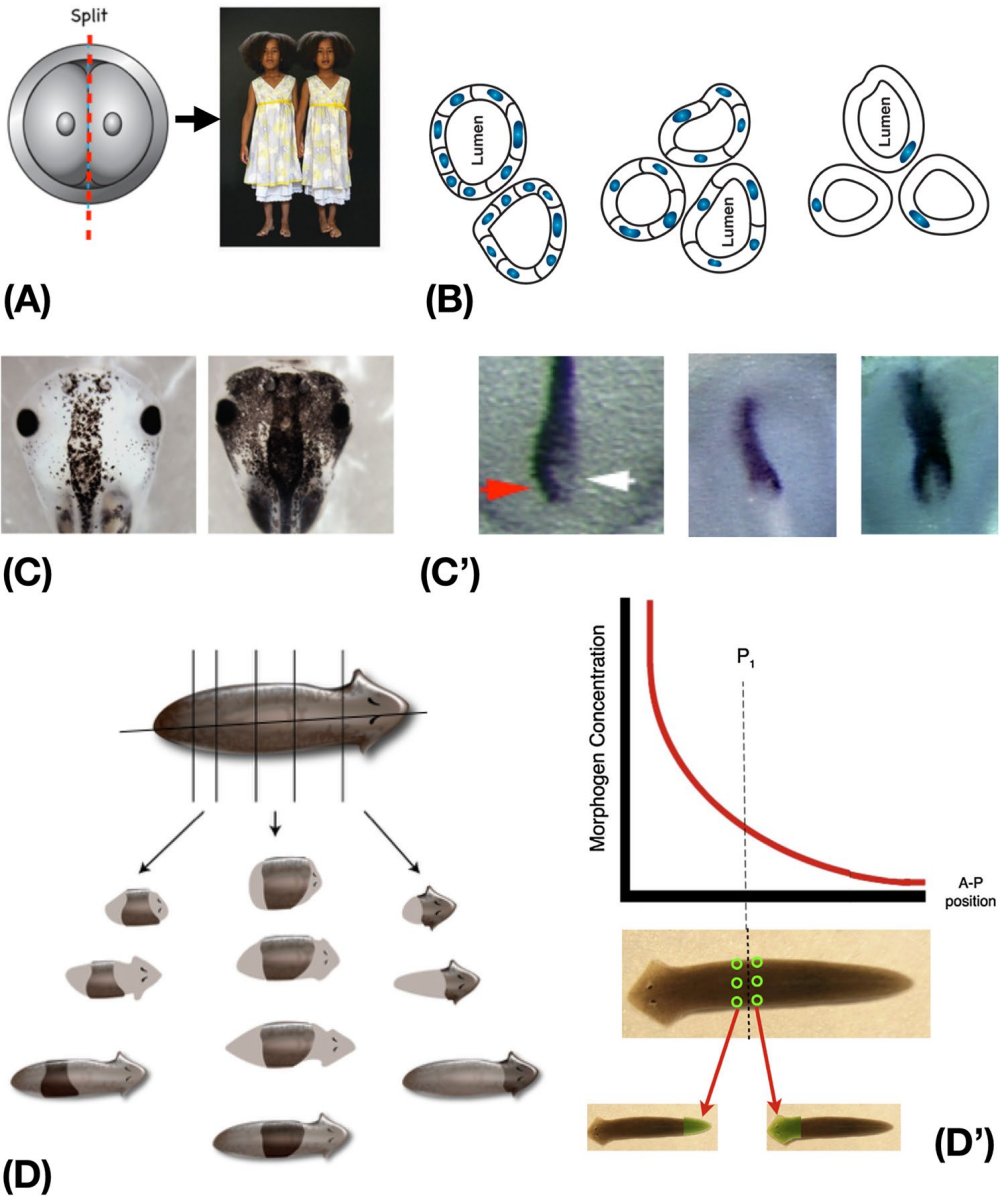
Multiscale control of morphogenetic competency. **A** Early embryos of many species, including humans, when split, result not in half-bodies but in monozygotic twins, illustrating the capacity for cells to recognize departures from the normal path and adjust accordingly. **B** Cells of the kidney tubule in newts not only make up for artificially enlarged sizes (using fewer cells to make the same size of tubule and newt) but in fact can harness diverse low-level molecular mechanisms to achieve their target morphology: when cells get truly huge, instead of cell:cell cooperative mechanisms, the system uses cytoskeletal bending to allow cells to wrap around themselves to achieve the same anatomical outcome. This illustrates top-down control across scales of organization, as also exploited in animal behavior. **C** Another aspect of multi-scale architecture is the ability of cells to make collective decisions. In the control of the melanocyte → melanoma transition in tadpoles (driven by a disruption of bioelectrical cues), stochastic behavior can be observed: some percentage of the animals in a given cohort stay normal (left side) and some become hyperpigmented (right side). However, this decision is always made at the whole animal level: each animal is either entirely normal or entirely hyperpigmented—regardless of the stochasticity, the bioelectric circuit enables the cells to in effect toss the same coin and make a system-level coordinated decision. The same is seen (**C’**) when left–right patterning cues are disrupted in early chick development. Here is shown the expression of the gene *Sonic hedgehog*, which should normally be expressed only on the left side of Hensen’s node (red arrow), not the right (white arrow). This can be randomized by various treatments to be right-sided (middle panel) or bilateral (right panel), but each side of the Hensen’s Node decides as a coherent system—speckling (decisions on individual cell level) is never seen. Each developmental domain makes a stochastic L vs. R decision as a unit, coordinating among all the cells as a tissue-level outcome. **D** In planaria, each fragment can regenerate an entire worm (a kind of holographic pattern memory). Critically, however, the anatomical decisions cannot be made locally according to any simple gradient scheme. When bisected, the anterior-facing and posterior-facing cells have radically different anatomical fates (building a head vs. a tail), but were direct neighbors before the cut (i.e., at the same position along the axis). What to do after injury cannot be decided by purely local cues, but must be derived by communicating with the rest of the fragment to determine where in morphospace they are located, and thus what must be done to reach the correct target morphology. Panel A photo by Oudeschool via Wikimedia Commons; other images used with permission from (Blackiston et al. 2011; Levin et al. 2019) and Jeremy Guay of Peregrine Creative

The deep conceptual and mechanistic parallels between behavior and morphogenesis (Grossberg 1978) suggest a research program that can catalyze novel progress in the life sciences: accessing multiple levels of anatomical control (Figs. 5,6). To truly understand the origin of cognition, and conversely, to deploy the insights of neuroscience for regenerative medicine and bioengineering, it is important to investigate the proto-cognitive capacities of morphogenesis, specifically, the range of behaviors and capabilities that the collective intelligence of body cells can deploy toward adaptive behaviors in anatomical morphospace (Figs. 7, 8). These range from purely emergent, hardwired morphogenetic cascades (corresponding to fixed, inborn instinctual behaviors) and complex, flexible ability to reach the correct target morphology despite novel circumstances (corresponding to a degree of intelligent problem-solving behavior). It is critical to recognize and study the ways in which morphogenesis is not simply an emergent result of local rules but has complex, multi-scale feedback mechanisms that implement a degree of goal-directed activity and problem-solving in a way that was not directly encoded by genome (a hallmark of cognitive mechanisms).

### Memory and target morphology

The most basic component of proto-cognitive capacities is that of memory. Morphogenetic memory is ubiquitous in, for example, regeneration. A salamander whose limbs, eyes, tail, or other organs are amputated will regrow exactly the right structure and stop all of the complex cell proliferation and remodeling activity when, and only when, a correct structure is complete (Harris 2018; Pezzulo and Levin 2016). While no individual cell knows what a finger is or how many a salamander is supposed to have, the tissue collective clearly has a setpoint for anatomical homeostasis that functionally guides morphogenetic behavior (memory) as an error minimization (i.e., objective function) process. This pattern memory can, like any good memory, be modified by experience (it is stable, but also labile to the right kinds of stimuli). For example, repeated amputation of axolotl limbs leads to the tissue habituating to the loss of limb and eventually giving up trying to re-grow (Bryant et al. 2017). Trophic memory in deer (Bubenik and Pavlansky 1965; Lobo et al. 2014) enables an ectopic branch in their otherwise stereotypical antler structure to be formed *year after year*, at a specific point of earlier damage, long after the damaged antler rack has been shed (a remarkable example of memory of position in 3D space, used to guide the growth of bone and nerve toward a new pattern, by cells at the scalp that have to regenerate the large structure every year).

### Re-writing morphogenetic memories: ontogenetic and evolutionary timescales

**Fig. 9:**
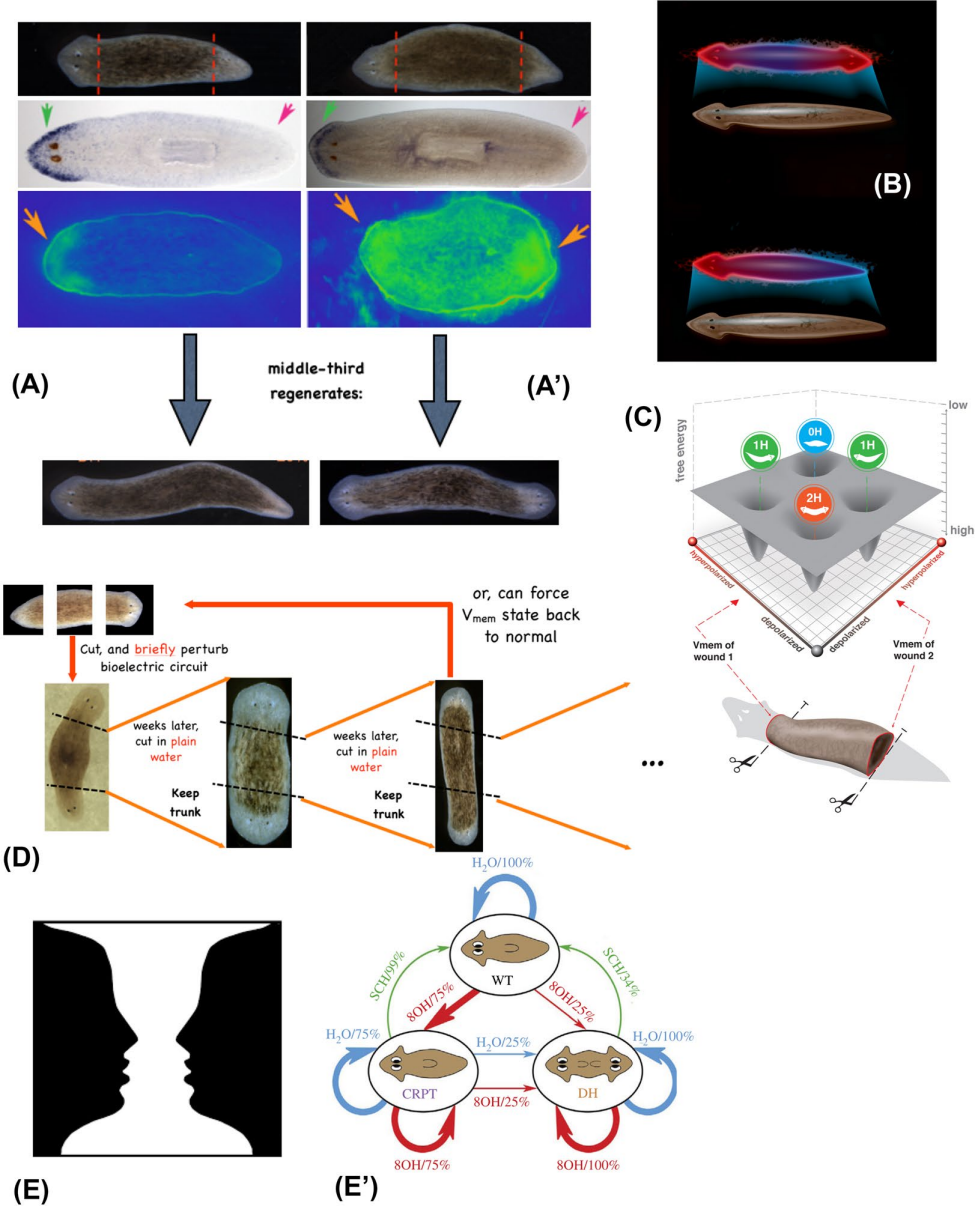
Morphogenetic memory can be re-written non-genetically. **A** Normal planaria (left column) exhibit anterior gene expression in the head, and when cut into three fragments, produce normal one-headed worms. In contrast (**A’**), planaria in which the normal bioelectric pattern has been re-written by brief pharmacological targeting of ion channels (blue panels, green indicates depolarized regions on the voltage map) give rise to two-headed animals. Note that the bioelectrical map shown is a map of the animal pre-cutting. **B** Thus, a normal planarian body can store one of 2 (at least) representations of what a correct planarian should look like. Much as in the nervous system, somatic bioelectricity enables changes in how systems navigate morphospace based on experience, not only genetic rewiring. **C** Bioelectric circuit dynamics enable the planarian fragments to navigate a morphospace which contains attractors for 0, 1, or 2 heads as the target state which each fragment seeks to achieve. **D** Ability to reliably reach the right morphogenetic state is indeed a kind of memory which is stable but re-writable. Two-headed animals continue to give rise to two-headed animals upon further rounds of amputation (deviation into an incorrect region of morphospace), without changing the genetics of the cells. The two-headed state can be reversed by the same kind of technique, targeting ion flux to re-set the target morphology representation back to a one-headed configuration. **E** Kind of perceptual bistability seen in ambiguous images (such as this famous “2 faces vs. vase” example) is also seen in morphogenetic systems: so-called Cryptic Worms have a destabilized target morphology memory, producing stochastically one-head or two-head forms upon each round of cutting. *WT* wild type, *CRPT* cryptic state, *DH* double-head. This ethnogram, applied to morphogenetic experiments, shows the transition probabilities when cut in water (H2O) or octanol (8OH, a gap junction blocker) or SCH28080 (SCH, a proton–potassium exchanger inhibitor). Images in panels **B**, **C** are by Jeremy Guay of Peregrine Creative. Others are used with permission from (Durant et al. 2017; Levin 2021a)

The ability of morphogenetic machinery to modify its outcome due to experiences can be externally controlled (Fig. 9), analogous to the inception of false memories in neuroscience contexts (Liu et al. 2014; Ramirez et al. 2013; Vetere et al. 2019). For example, the number of heads in a regenerating planarian flatworm fragment is set by the state of a bioelectric circuit in the tissue (Beane et al. 2011). It has a built-in default of 1 (much like default, innate behaviors of brainy creatures), but is re-writable. Transient modification of the bioelectric state is remembered by the circuit, which can be experimentally re-set to read “2”: worms regenerating from such fragments indeed have two functional heads, one at each end (Durant et al. 2019; Oviedo et al. 2010). Remarkably, when cut again in multiple subsequent rounds of regeneration (with no further manipulation), the fragments continue to result in two-headed animals. This shift in the target morphology for regeneration is permanent (unless the animal’s bioelectric pattern memory is reset back to a one-headed state by an experimental manipulation). Thus, much like in nervous systems, the architecture underlying memory in this space consists of electrophysiological hardware with a highly reliable default behavior (guiding to the one-head region of morphospace) but also the capacity to be re-written toward other states, which ultimately feed into changes in gene expression and long-term changes in cell properties (Bischof et al. 2020; Pai et al. 2016; Pietak et al. 2019). The standing pattern of resting potential differences which instructively determines the number of heads that will be built is quite literally the memory of the collective intelligence of the body—a bioelectric state that guides its behavior in anatomical morphospace and can be modified by experience in physiological space. This system matches all the key criteria for memory: it is long-term stable, yet labile (rewritable), enables discrete behaviors induced by stimuli, and can even be latent until needed (see below).

While the genome encodes the hardware (the cellular affordances, such as ion channels), the actual outcome is the result of context- and experience-sensitive electrophysiological software that this hardware supports (see (Bongard and Levin 2021; Bongard and Levin 2023; Nicholson 2014) for an in-depth discussion of the machine analogy in biology). The question, of how many heads a given cellular collective will produce, is, surprisingly, not directly encoded in its genome because exactly the same, wild-type set of cells can produce 1-, 2-, or 0-headed planaria depending on their history. The same is true of a single embryonic blastoderm, which normally produces 1 “embryo”, but can produce multiple conjoined individuals if the cell:cell communication is temporarily disrupted (Lutz 1949). Of course, despite its essentially epigenetic [in the broad, original sense of the word (Ginsburg and Jablonka 2009; Jablonka 2012; Jablonka and Lamb 1995; Jablonka and Raz 2009)] nature, the bioelectrical control system works together with genetic information. Evolutionary tweaks of ion channel properties that eventually lead to genetic assimilation of bioelectrically induced phenotypes is likely an important aspect of the evolution of body plans and other morphological features, much as the Baldwin effect is thought to be a key dynamic for the role of cognition and learning in evolutionary lineages (Baldwin 1896).

### Reprogrammability, external manipulation, and representation

Three especially interesting analogs with behavioral cognition have been shown in this system. First, the advantage of such a system is the same as for neuro-cognitive architectures: it allows behavior modes that are not specifically genetically encoded, broadening the range of adaptive maneuvers that can occur in novel circumstances. The disadvantages are similar too: much as the mechanisms of brain function can be exploited by parasites using chemical (da Silva and Langoni 2009) or linguistic hijacking (Kurbel and Kurbel 2019; Panchal and Jack 2022), bioelectric control mechanisms also subject morphogenetic plasticity to potential hijacking (Williams et al. 2020). For example, much as host behavior can be specifically altered when parasites manipulate neurobehavioral systems to their own advantage (da Silva and Langoni 2009; Vyas and Sapolsky 2010; Webster and McConkey 2010), the planarian morphogenetic control system too can be hijacked not only by bioengineers (as described above) but also by commensal bacteria which can over-ride the default system and lead to two-headed regeneration (Williams et al. 2020).

Second, two-headed patterns can exist in a one-headed animal (prior to injury). This means that, analogously to the brain’s capacity for counterfactual thinking, a single physical structure [normal planarian body, with normal gene expression profiles (Durant et al. 2017)] can encode multiple memories of what the correct morphogenetic goal state is. In the case of a two-headed pattern in a one-headed host, this means that the bioelectrical pattern is not a read-out of what the anatomy is currently, but rather a representation of what it *should be*, weeks later, following regeneration *if* the animal is injured. It is in effect then, a counterfactual memory that is tied not to current state but to a possible future. This primitive system is one way to begin to think about the origins of mental time travel that brains enable, with all of its obvious adaptive advantages. It should be emphasized that the traditional lens of viewing development as an emergent property of a dynamical system that simply ends up in specific states does not facilitate the kind of research direction that enabled this new biology to be discovered (and in general, the open-loop emergence paradigm makes it very hard to see how novel outcomes can be rationally controlled). In contrast, it is specifically the search for an encoded representation as a pattern memory that guides morphospace navigation that led to the methods to directly observe (Figs. 5A,9A) and edit this information structure leading to permanent, top-down changes of the target morphology.

Finally, planarian regeneration offers an example of bistability (Pezzulo et al. 2021)—a common feature of perceptual systems with top-down control. Exposure to ion channel drugs (a physiological experience akin to electrical signals from a retina or other sense organ, not a genetic change) can place the bioelectric circuit into a state in which it cannot decide between two outcomes and flips back and forth stochastically at each regenerative event (recall of target morphology). Fragments from such “cryptic” worms (Durant et al. 2017) will regenerate as one-headed or two-headed upon each cut, randomly. These fascinating parallels between key features of cognitive and morphogenetic systems shed light on the origins of behavior and offer a simplified context in which to probe the complex aspects of conventional cognition.

### Creative problem-solving by adaptive morphogenesis: plasticity, not just emergent complexity

Another interesting aspect of cognition is creative problem-solving: the ability of systems to achieve goals in novel circumstances or in ways that are different from their default [this is William James’ (James 1890) definition of *intelligence*—the ability to achieve the same goals by different means]. This definition is suitably cybernetic in its framing (emphasizing substrate-independent function and a degree of autonomous agency, not a specific neural architecture of phylogenetic position). At the same time, it reminds the experimenter that the formalization of goals and behavior are not absolute but observer-dependent: claims of goal-directedness (Clawson and Levin 2022; Levin 2023) must include a specification of a proposed problem space, a proposed ensemble of states that are hypothesized to be the goal states, and testable claims of how much and what kind of competencies to reach the goal can be expected when that system is stressed away from its normal course of events (Fields and Levin 2022; Levin 2022).

One example of this plasticity of morphogenetic behavior (expanding on the default genetically encoded repertoire) is the fact that temporary exposure to a blocker of electrical synapses (i.e., an anesthetic) causes planarian fragments to create the heads of *other species*, with no genetic change required (Emmons-Bell et al. 2015; Sullivan et al. 2016). The bioelectric network incorrectly navigates to additional attractors in morphogenetic space that are normally used by species 100–150 million years of evolutionary distance—all without any genetic change needed (much like nervous systems allow an animal to dynamically remember or envision novel, distant scenarios without the need for genetic change to its brain hardware to enable each new thought). Much as with human patients exiting general anesthesia, who often hallucinate for a time while the brain network is finding its way back to the correct pre-anesthetic state, but then (usually) recover their correct personal identities, planaria that build the wrong species’ head shapes eventually remodel back to normal.

The robust regulative properties of bodies strongly emphasize the system’s ability to solve novel problems. For example, early mammalian embryos cut in half do not form two half-bodies (as any hardwired, purely emergent system would). Instead, each side recognizes the damage, makes up for it exactly, and creates one of a pair of monozygotic twins. Perhaps even more remarkable is the case of newt kidney tubules (Fankhauser 1945). By default, they consist of 8–10 cells in cross section. However, if the cells of the early embryo are artificially made to be larger, fewer cells will be used, resulting in the same (normal) tubule diameter and overall body size. Remarkably, this can be pushed to a fascinating extreme: if the cells are made to be enormous, a single cell will bend around itself, producing the normal size tubule diameter. This example illustrates not only the ability to reach the same anatomical state despite diverse and novel starting conditions with no need for periods of lengthy adaptation, but also the startling ability to call up diverse molecular mechanisms (cell:cell communication in normal conditions, but cytoskeletal bending in the case of huge cells) as needed in the service of a large-scale anatomical goal. This is an example of top-down control, in which lower level mechanisms are activated based on high-level needs—an essential feature of nervous system architectures which enables the same phenomenon in behavioral space (e.g., executive-level decisions and high-order goal states that filter down to control of actin dynamics in muscle cells as implementation machinery).

Another example of cellular plasticity beyond the default behaviors of the genetically specified hardware is that of Xenobots—the proto-organisms that spontaneously form from dissociated frog embryo skin cells (Blackiston et al. 2021; Kriegman et al. 2020, 2021a). These living forms are motile (via cilia normally used to distribute mucus along the frog’s skin) and self-directed, performing a variety of spontaneous behaviors. The most remarkable novel behavior is that of kinematic self-replication: Xenobots build copies of themselves by rearranging loose cells provided to them in the medium. This is Von Neumann-style replication that is not, to our knowledge, used by any other species on Earth. Having been deprived of the normal ability to reproduce, Xenobots arrive at a novel solution within 48 h of being created for the first time (they have no history of evolutionary selection to be a good Xenobot). These examples of real-time morphological and functional adaptation, including classic ones, such as Slijper’s Goat (Slijper 1942), which acquired, in its own brief lifetime and not millennia, the body structures needed for upright bipedal walking, reveal the prodigious capacity for morphogenesis to enact creative solutions to novel problems using the same hardware. This is another essential hallmark of cognitive systems.

Thus, coherent, effective organisms form despite not being able to count on having the right number of cells, cells of the expected size, or even the same number of chromosomes (in the case of planaria and polyploid newts). This ability to handle novelty, not only in its external environment but also in that of its component parts, is the envy of the robotics and AI communities (Aubin et al. 2022; Bongard and Levin 2021; Kriegman et al. 2021b). The on-the-fly competencies of the morphogenetic control system offers evolution the same thing that nervous systems eventually offered: the ability to not over-train on evolutionary priors and instead generate problem-solving machines. Much like with behavior, it is impossible for evolution to foresee all of the novel circumstances that organisms will be required to deal with [well beyond the handful of possible environments usually studied in phenotypic plasticity and epigenetic controls (Fraebel et al. 2020; Santos et al. 2015; West-Eberhard 2005a, b)]. The amazing inter-operability of life [e.g., chimeras, bio-tech hybrids, etc. (Clawson and Levin 2022)] is a testament to the fact that life uses a “play the hand you’re dealt” system. It produces cellular collectives which carry out, not a rote set of steps, but rather a suite of second-order functions [such as active inference (Pezzulo et al. 2015; Pezzulo et al. 2018a, b) and other computational tasks] to achieve coherent function in a wide range of changing and unpredictable scenarios, both with respect to environment and also its own parts.

### Navigation: an invariant between morphological, behavioral, and other spaces

One formalism for understanding these capacities, and for learning to rationally manipulate them, is that of navigation (Fields and Levin 2022). Both behavioral and morphogenetic competencies can be modeled as various policies for navigating problem spaces. For example, during metamorphosis, the tadpole of *Xenopus laevis* can reach the correct frog face region of morphospace not only from its default starting position (a normal tadpole configuration), but also from a wide range of scrambled configurations (Vandenberg et al. 2012)—using new paths through that morphospace that nevertheless end up in the same goal region (Friston et al. 2015; Pezzulo and Levin 2016). Neural wiring does this as well, finding new paths to achieve functional network architecture in mutant mice (Little et al. 2009). As with certain animals that have automated, built-in behavioral repertoires, there are embryos (e.g., the nematode *C. elegans*) whose development seems largely hardwired. However, the vast majority of model species appear to use a combination of default modes and ability to improvise.

The full range of cognitive capacities for navigation of morphospace has only begun to be investigated—it is unclear how many of the advanced concepts from cognitive science (place cells, path planning, etc.) will become relevant. Moreover, these concepts in neuroscience are themselves in flux (Keijzer and Arnellos 2017; Keijzer 2017; Levin et al. 2021; Lyon et al. 2021; Pinotsis and Miller 2022). Given that cognitive capacities are present very widely across the biosphere, it is likely that advances in developmental bioelectricity may help to scaffold our understanding of neural systems and behavior.

A number of additional competencies (beyond the ability to achieve the target morphology despite modification to starting position or internal structures) have been found. For example, when an ectopic lens (in tail tissue) is induced by bioelectric modulation, it is sufficient to misexpress the channel only in a small subset of the needed cells: once their organ-level goal is specified, these cells will secondarily *recruit* other (un-modified) host cells as needed to achieve the critical mass needed to produce a normal-sized lens. While the engineer re-specifies the morphospace target region for a set of cells via a simple trigger, the necessary downstream modules (size control, substructure such as lens/retina/optic nerve placement, etc.) are automatically activated and do not need to be micromanaged (Gallistel 1980; Powers 1973). This modular control architecture, implemented by bioelectric circuits that set properties of a developmental compartment, is fundamental to the action of the brain (Bizzi et al. 1995; Callebaut et al. 2005; Levin and Yuste 2022), and in both contexts provides an interesting counterbalance with mechanisms of global integration. More specifically, this kind of ability of the collective to ascertain needs and modify the behavior of the right number of components is seen across swarm intelligences beyond morphogenesis, from that of ants recruiting conspecifics to a task (Burchill et al. 2022; Collignon et al. 2014; Wilson 1980), to neurons being recruited according to cognitive load (Bryer et al. 2013; Rossi et al. 2012).

#### E pluribus unum

The mechanisms that enable collectives to make high-level decisions and deploy their components as needed (deform the action space of subunits toward the goals of the collective in a new problem space) are only beginning to be understood. However, those dynamics are likely related to two key properties of the gap junctions common to morphogenetic and neurocognitive systems (Peracchia 2004; Trosko 2007). First, as with ion channels, they are themselves voltage-sensitive valves, enabling feedback loops and historicity. Second, by providing direct connections between the intracellular milieus of cells, they provide an “owner wiping property” for stress signals and other molecular traces of experience: cells cannot tell whether a given memory molecule (e.g., calcium flux) belongs to itself or its neighbors. This leads to partial erasure of individual identity for cells in a network, and enables a “collective” to scale up the measurements, stored goal states, and actions toward the emergence of larger scale agents operating in new spaces. Thus, while not necessarily an essential component of cognition in every possible living system (e.g., exo-biological contexts), bioelectric networks have been exploited extensively by life on Earth to provide the integrated computations needed to scale the homeostatic competencies of the most humble self-reproducing units into agents with flexible, highly adaptive behaviors in complex problem spaces.

## Interfacing with the collective intelligence: from evolutionary perspectives to biomedicine

Bioelectricity is fundamentally a mechanism to scale computation. While bioelectric states do control cell-level properties, such as plasticity, proliferation, differentiation, etc. (Levin 2021a), the real power in this system is in determining large-scale behaviors at the tissue and organ level (Harris 2021; Levin and Martyniuk 2018). It has been shown to control size (Daane et al. 2018; Perathoner et al. 2014; Yi et al. 2021), organ identity, and whole body axes (Levin 2021a). A critical (and brain-like) aspect of bioelectrical networks is the hierarchical organization of functionality and the association of complex morphogenetic activity with simple stimuli (triggers). Much like the central nervous system (CNS) allows complicated multi-step behaviors to be triggered by a low-information content stimulus, a brief and transient bioelectrical signal can induce whole eyes (Pai et al. 2012) and appendages (Adams et al. 2007; Tseng et al. 2010) in which all the internal details are handled autonomously.

This key feature is currently beginning to be explored for regenerative medicine applications. For example, amphibian tails (including spinal cord) and limbs can be induced to regenerate by specific bioelectric states triggered by ion channel drugs or optogenetic stimulation (Adams et al. 2013; Tseng et al. 2010). Severe brain defects in the frog model, induced by chemical teratogens or mutation of a critical neurogenesis gene (Notch), have been rescued by reinforcing appropriate bioelectrical signaling (Pai et al. 2020, 2015; Pai and Levin 2022). Especially in the case of Notch mutation-induced malformations, the fact that such fundamental hardware defects can be in effect resolved “in software” (by drugs modulating *V*_mem_ to force the correct pattern) suggests the potential power of these interventions for addressing defects and traumatic injury for which we have few viable treatments today. This potential is akin to how behavioral accommodations can often make up for even severe structural defects (Slijper 1942). Similarly, tumorigenesis has been shown to be controllable by modulation of bioelectric state—normalizing cancer by reconnecting cells to the electrical network that harnesses them toward adaptive tissue homeostasis (Chernet et al. 2016, 2015; Chernet and Levin 2013b), a promising alternative to current toxic chemotherapy approaches.

Efforts to control the information processed by bioelectric networks toward particular outcomes are now being guided by computational tools at multiple levels of organization, applying Marr’s (Marr 1982; Peebles and Cooper 2015) taxonomy to control of body form just as neuroscience addresses the problems of behavior at levels from that of synaptic proteins to psychiatric therapies (Adams et al. 2016; Friston et al. 2014; Wang and Krystal 2014). However, it is likely that this work has only begun to scratch the surface of what is possible. Biomedicine today remains largely focused on the hardware—micromanaging genes and pathways toward desired functionality— and has not yet internalized the fundamental wisdom of the multi-scale approach in the neuro-behavioral sciences. The research summarized above suggests that behavior-shaping and training paradigms for cells and tissues will likely enable much greater control of morphology, gene expression, and pathway function than can be realistically achieved bottom-up (just as training animals for specific behaviors is more efficient than attempting to run their muscles directly like puppeteers). Transformative improvements in capabilities in bioengineering and regenerative medicine are within reach, if we learn to appropriate as much as possible of the multi-scale wisdom of neuroscience and generalize it beyond neurons.

## Conclusion

The impacts of behavioral neuroscience, and of its advances in explaining cognitive and proto-cognitive capacities, lie well beyond classical neural cells. Understood broadly, developmental bioelectricity provides an entry-point into unifying adaptive “behavior” and problem-solving intelligence in diverse spaces in a way that makes it natural to think of plant, microbial, and even synthetic life (Baluška and Mancuso 2012; Baluška et al. 2022; Baluška and Reber 2021a, b; Bassel 2018; Calvo et al. 2020, 2017; Debono and Souza 2019; Martinez-Corral et al. 2019; Prindle et al. 2015; Reber and Baluška 2021; Schofield et al. 2020; Solé et al. 2016; Souza et al. 2017; Urrios et al. 2016; Yang et al. 2020) using the same conceptual tools from behavioral and physiological sciences. The on-going debate around representation and morphological computation is likewise being enriched by data in this field (Keijzer 1998, 2001).

The remarkable fundamental mechanism that enables a true emergent collective intelligence—a mind (at whatever scale of sophistication)—is ancient, and is also responsible for the plasticity and robustness of morphogenesis. Evolution re-used some of the same computational strategies, for binding competent signaling subunits into networks with memory and problem-solving capacity, to navigate a diverse set of spaces (physiological, anatomical, behavioral, and linguistic). Bioelectricity offers a tractable and powerful entry-point into understanding this process, because it serves as the cognitive medium of collective intelligence—whether of neurons in the brain, or of cells in a body trying to achieve anatomical outcomes. Thus, firm conceptual (and disciplinary) distinctions between the science of the brain and those of the body are largely artificial hold-overs from past limitations of technology and evolutionary understanding, and are increasingly being erased (Anderson et al. 2012; Beer 1995; Pezzulo and Levin 2015; Pfeifer et al. 2007; Willems and Francken 2012). Future work will address the mechanisms by which developmental bioelectricity sets up the structure of the CNS (Pai et al. 2015) and the ways in which neural signals control morphogenesis (Belgacem and Borodinsky 2015; Borodinsky et al. 2012; Herrera-Rincon and Levin 2018; Herrera-Rincon et al. 2017); beyond the molecular mechanisms linking these two systems, there is also much opportunity for unification into a single underlying conceptual architecture.

**Fig. 10:**
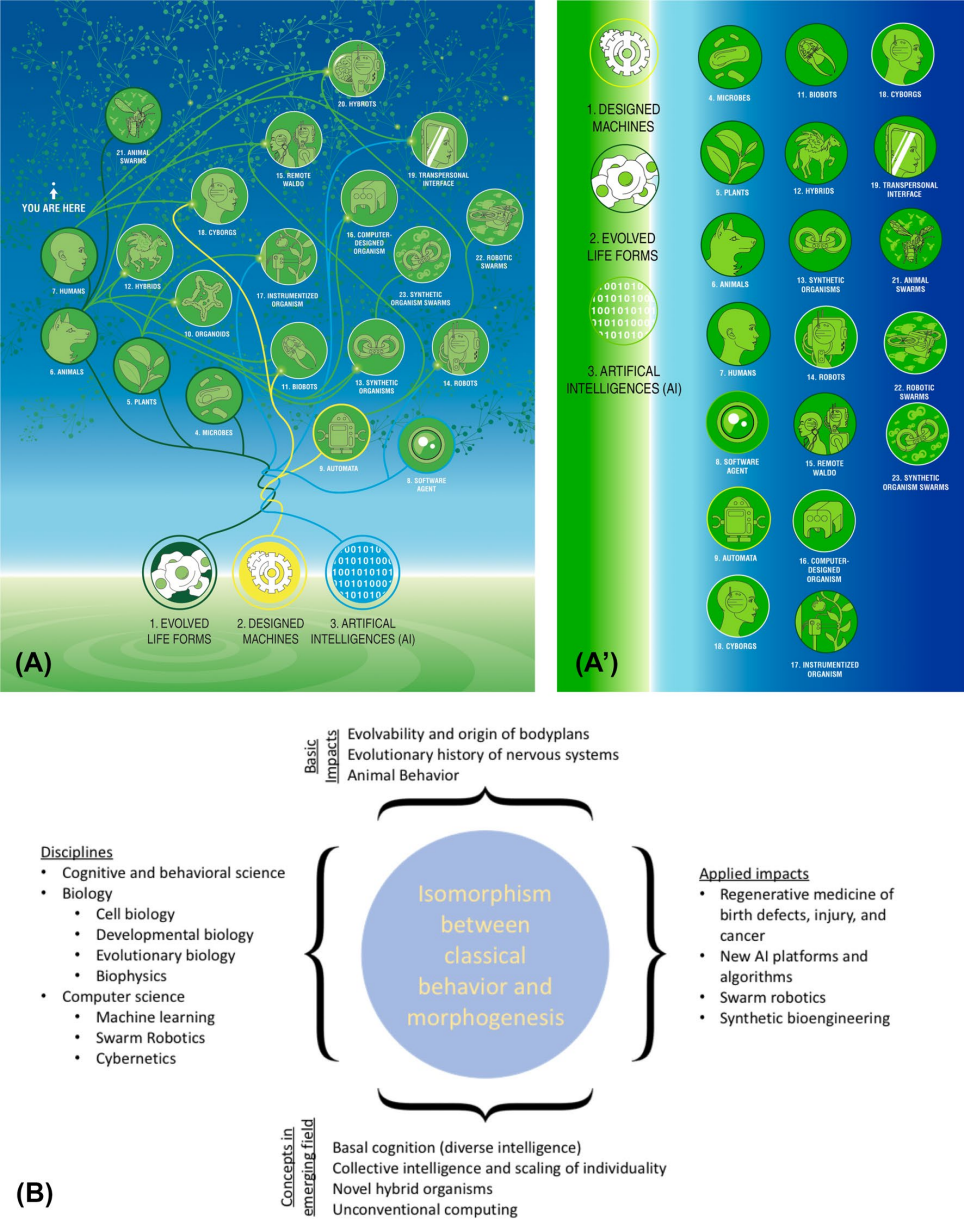
Future of the diverse intelligence field. **A** Because of the deep ability of living cells to form collectives with adaptive functions under novel circumstances, many diverse combinations of evolved material (cells, tissues), designed material (engineered smart materials or implants), and software (AI systems) are viable. The future is likely to include a massive number of highly diverse agents with various degrees of behavioral competency (**A’**: hybrots, cyborgs, biorobots, etc.) which will be the subject of an expanded field of animal behavior research that is not limited to the set of natural animals here on Earth. **B** Mindmap of the emerging field at the intersection of the sciences of animal behavior and morphogenetic control, showing the many disciplines whose ideas feed into this new consilience, and the deep concepts and practical impacts that progress in this field will have. Images in panels **A** and **A’** are by Jeremy Guay of Peregrine Creative

Evolution exploits the generic computational properties of such networks (learning, generalization, counterfactual memories, representation, distributed control, etc.) at many scales, building flexible problem-solving engines instead of fixed solutions to specific environments (Moczek et al. 2011; Oyama 2000; Sultan et al. 2022). While bioelectricity is an especially convenient modality, proto-cognitive capacities (such as learning) are found also in biochemical networks and biomechanical networks (Biswas et al. 2021; Stern et al. 2020a, b; Watson et al. 2010). Emerging frameworks that focus on a ubiquitous multi-scale competency architecture, where each layer has some degree of behavioral and proto-cognitive capabilities that await discovery, are already generating new capabilities and driving novel research programs (Aubin et al. 2022; Bongard et al. 2006; Davies and Levin 2023; Kriegman et al. 2021b; Levin et al. 2017; Merrild and Rasmussen 2018; Pezzulo 2020; Pezzulo et al. 2021; Pezzulo and Levin 2015, 2016, 2018; Pfeifer et al. 2005; Taylor et al. 2016). The implications of this approach (Fig. 10) range far beyond behavioral and developmental sciences, to encompass synthetic bioengineering of novel cognitive life forms (Clawson and Levin 2022; Ebrahimkhani and Levin 2021; Kamm and Bashir 2014) and related fields, such as robotics and AI. All of these disciplines are beginning to intersect in the exciting emerging field of diverse intelligence research, which will not only provide numerous useful applications in engineering and biomedicine, and help to understand our evolutionary history, but most importantly, will shed light on the deepest philosophical problems of the origin and nature of possible cognitive Selves.

## Data Availability

Not applicable.
